# Idebenone increases chance of stabilization/recovery of visual acuity in *OPA1*‐dominant optic atrophy

**DOI:** 10.1002/acn3.51026

**Published:** 2020-04-03

**Authors:** Martina Romagnoli, Chiara La Morgia, Michele Carbonelli, Lidia Di Vito, Giulia Amore, Corrado Zenesini, Maria Lucia Cascavilla, Piero Barboni, Valerio Carelli

**Affiliations:** ^1^ IRCCS Istituto delle Scienze Neurologiche di Bologna UOC Clinica Neurologica Ospedale Bellaria Bologna Italy; ^2^ Department of Biomedical and Neuromotor Sciences (DIBINEM) University of Bologna Bologna Italy; ^3^ IRCCS Ospedale San Raffaele Milan Italy; ^4^ Studio Oculistico d’Azeglio Bologna Italy

## Abstract

We previously documented that idebenone treatment in *OPA1*‐Dominant Optic Atrophy (*OPA1*‐DOA) led to some degrees of visual improvement in seven patients. We here present the results of a cohort study, which investigated the effect of off‐label idebenone administration in a larger *OPA1*‐DOA group compared with untreated patients. Inclusion criteria were: *OPA1*‐DOA clinical and molecular diagnosis, baseline visual acuity (VA) greater than/equal to counting fingers and treatment duration greater than 7 months. We found a significant difference between the last visit and baseline VA in favor of stabilization/recovery in idebenone‐treated as compared to untreated patients. This effect was retained after controlling for confounders.

## Introduction

The results of a double‐blind, placebo‐controlled, randomized clinical trial,^1^ and a large retrospective survey of patients affected by Leber’s hereditary optic neuropathy (LHON)^2^ converged on showing that idebenone increases the rate of visual recovery, in particular when given at early stages after onset and for a prolonged time. In 2013, we reported a pilot study on seven patients with another inherited optic neuropathy due to mitochondrial dysfunction, dominant optic atrophy (DOA) associated with *OPA1* haploinsufficiency heterozygous mutations, who were treated for at least 1 year with idebenone.^3^ Many common features across LHON and DOA prompted us to use off‐label idebenone in DOA. First, despite the subacute natural history of LHON as opposed to the congenital or infantile‐onset and slow, relentless progression of DOA, in both diseases, the pattern of axonal neurodegeneration in the optic nerve is similar.^4^, ^5^ In fact, in both disorders, the small axons of the papillomacular bundle are affected first and more severely, leading to the temporal pallor of the optic disc at fundus examination, central scotoma at visual fields and loss of central vision with a consistent drop in visual acuity.^4^, ^5^ Second, the hallmark of mitochondrial dysfunction in LHON is linked to an obvious primary dysfunction of complex I,^6^ whereas *OPA1*‐linked DOA is characterized by defective mitochondrial fusion and cristae derangement, which in turn lead to defective oxidative phosphorylation with reduced ATP synthesis driven by complex I substrates.^7^ Idebenone shuttles electrons directly to complex III, thus bypassing complex I, and acts as antioxidant.^8^ In both diseases, there is also a well‐documented propensity to a chronic increase of reactive oxygen species production.^4^, ^5^ The results of the seven idebenone‐treated *OPA1*‐mutant DOA (*OPA1*‐DOA) patients pointed to a possible beneficial effect of idebenone therapy documenting the improvement of VA in these patients.^3^

After this pilot report, a larger group of DOA patients carrying either *OPA1* mutations leading to haploinsufficiency or missense point mutations have been treated with idebenone. Thus, through this observational cohort study, we aimed at investigating the effect of off‐label idebenone administration on visual outcome in *OPA1*‐DOA patients compared to untreated *OPA1*‐DOA individuals, considered as controls.

## Materials and Methods

This study has a historical cohort design and follows the STROBE guidelines.^9^ All subjects gave written informed consent for the collection of clinical data, data analyses, and publication. The study was conducted in agreement with the Declaration of Helsinki and approved by the local ethics committee (EC#121/2019/OSS/AUSLBO).

Patients satisfying inclusion criteria for a molecular and clinical defined *OPA1*‐DOA diagnosis were enrolled and divided in those who were treated with off‐label idebenone for at least 7 months between April 2007 and April 2017, compared with those untreated (controls). Similarly to our previous retrospective analysis of idebenone use in LHON^2^ and to the pilot study with a small DOA case series in 2013^3^, the treated patients received idebenone under the Italian regulation for off‐label drug administration,^10^ in a similar range of dosages (135–675 mg/day). The vast majority of patients were treated or started treatment before idebenone approval for LHON in 2015 (https://www.ema.europa.eu/en/medicines/human/EPAR/raxone#authorisation-details-section). The dosage in individual cases was modulated to avoid the occurrence of specific clinical side effects or blood exam abnormalities (headache and insomnia, weight changes, age, neutropenia, alteration of liver function indices, hypercholesterolemia, gastrointestinal disturbances), thus adjusting to maximal dosage without side effects, as required for off‐label drug administration. Patients with evidence or previous history of glaucoma or with any optic neuropathy other than DOA, and with a baseline best‐corrected‐visual acuity (VA) less than counting fingers (CF) were excluded.

We identified 87 *OPA1*‐DOA patients and stratified them into 37 untreated subjects and 50 idebenone‐treated.

**Figure 1:**
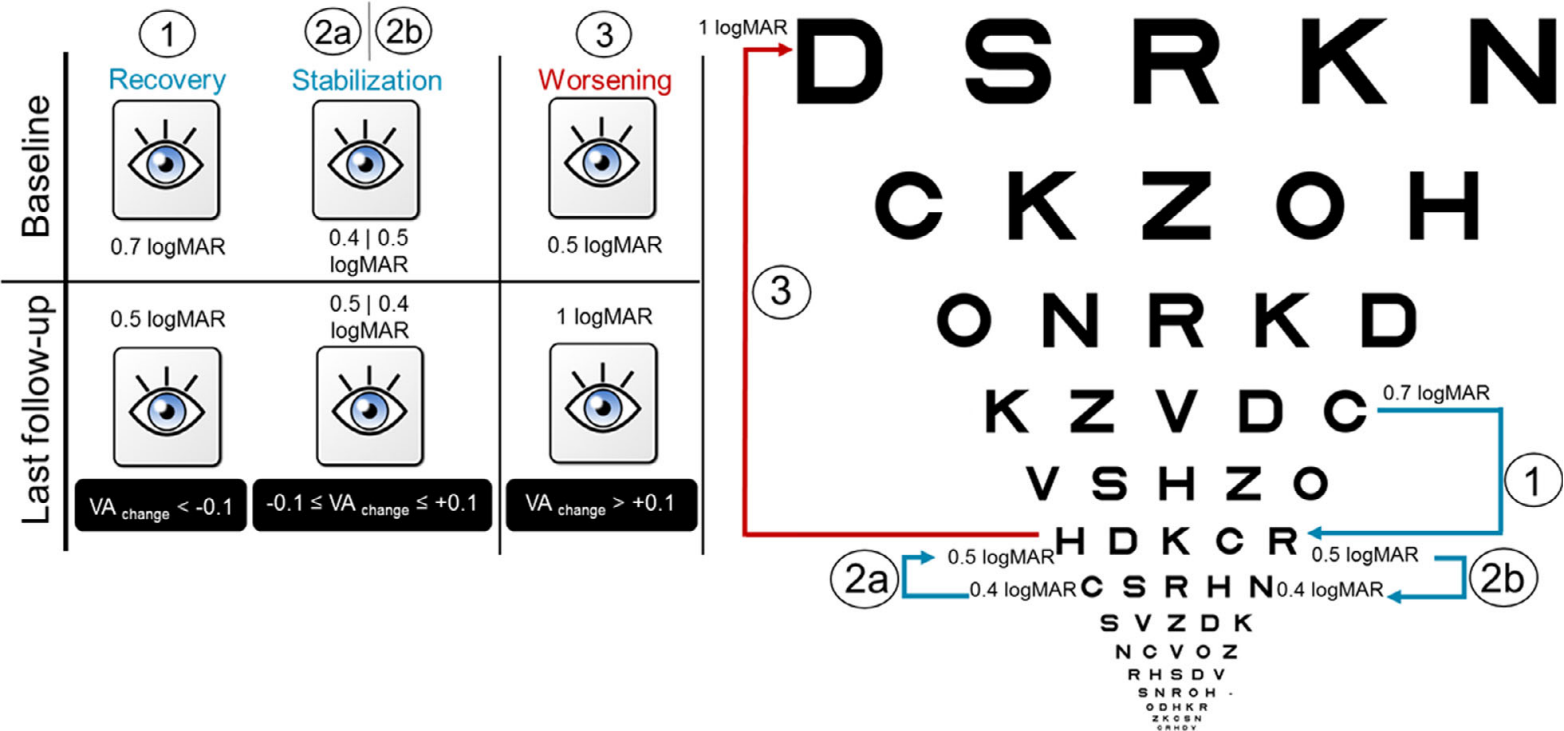
*OPA1*‐DOA visual acuity outcome between baseline and last follow‐up visits. Three possible scenarios of VA outcome are shown by way of example: recovery (1); stabilization (2a and 2b) and worsening (3). The VA outcome of interest for statistical analysis was the VA stabilization/recovery, defined as a best‐corrected‐visual acuity change (VA change). VA = best‐corrected‐visual acuity in logMAR unit.

The current knowledge of the natural history of *OPA1*‐DOA is characterized by a relentless visual loss progression without spontaneous recovery.^4^, ^5^, ^11^, ^12^ We evaluated the best‐corrected‐visual acuity change (VA change) between the baseline and the last follow‐up. We defined VA stability as changes ± 0.1 logMAR (logarithm of the minimum angle of resolution), whereas recovery for changes <−0.1 logMAR and worsening for changes> 0.1 logMAR (Fig. 1). On the basis of this primary outcome, patients were subclassified into 2 groups:

‐ stabilization/recovery;
‐ worsening.

For treated patients, baseline was considered as the last VA before the start of idebenone therapy, whereas in untreated baseline was the first available VA.

For statistical analysis, eyes were classified into the best‐ and worst‐seeing based on baseline visual examination. The pre‐defined algorithm for determining the best‐seeing eyes was the following:

1. logMAR VA: the subject’s eye with lower logMAR VA was the better‐seeing eye. If both eyes had an equal logMAR acuity, the following criterion was used;
2. Mean Deviation (MD) for VF (Visual Field, 30‐2 or 24‐2);
3. if the eyes were equal based on criteria 1 and 2, the best‐seeing eye was randomly selected.

We followed a “one‐eye” approach by evaluating the best‐seeing eye as the one assumed to have more benefit from the idebenone therapy. We also assessed the worst‐seeing eye, to check for the degree of concordance of the two eyes as non‐independent variables.

Chi‐square, Wilcoxon, Mann‐Whitney U‐ tests were used to compare variables among groups. We performed univariate and multivariate logistic regression analyses to study the association between idebenone administration (exposure), the outcome of interest (VA stabilization/recovery) and confounding factors (gender, *OPA1* mutation type, baseline VA, baseline age, observation time). Two‐sided *P*‐values and 95% CIs are presented. For statistical analyses, SPSS (SPSS Inc., IBM, Chicago, IL, USA) and Stata SE (StataCorp, College Station, TX, USA) softwares were used.

## Results

In this study we included 87 patients from 69 unrelated pedigrees (Table 1). Demographic and clinical characteristics were comparable between groups (Table 1: sex, *P* = 0.69; *OPA1* mutation, *P* = 0.47; age at baseline, *P* = 0.85; observation time, *P* = 0.15; baseline VA for best‐seeing eyes, *P* = 0.26; baseline VA for worst‐seeing eyes, *P* = 0.26). Most patients (*n* = 24, 48%) took 540 mg/day of idebenone, ten 270 mg/day, ten 405 mg/day, five 675 mg/day, and only one 10‐year‐old patient received 135 mg/day. According to the established criteria we classified 74 eyes as stable/recovery and 13 as worsening. Considering the 74 stable/recovery best‐seeing eyes, 94.5% of the corresponding worst‐seeing eyes were stable/recovery as well (data not shown).

**Table 1:** Demographics and clinical features of *OPA1*‐mutant DOA patients.

	Untreated	Treated	*P*‐value*, 1
Patients	37 (42.5%)	50 (57.5%)	
Gender			
Male	23 (62.2%)	29 (58%)	0.69
Female	14 (37.8%)	21 (42%)	
*OPA1* mutation			
Haploinsufficiency	25 (67.6%)	32 (64%)	0.47
Missense mutation	10 (27%)	18 (36%)	
NA	2 (5.4%)	—	
Age at baseline	29.4 ± 16.8 (14.6–43.2)	30.5 ± 17.6 (14.4–46.1)	0.85
Observation time (years)	3.4 ± 2.5 (1.4–5.5)	4.2 ± 2.3 (1.9–6.2)	0.15
Best‐seeing eye VA at baseline (logMAR)	0.58 ± 0.42 (0.22–0.90)	0.7 ± 0.42 (0.3–1)	0.26
Worst‐seeing eye VA at baseline (logMAR)	0.7 ± 0.48 (0.3–1)	0.8 ± 0.46 (0.5–1)	0.26

Values are given as *n* (%) or mean ± standard deviation (interquartile range, Q1–Q3).

NA, not applicable; VA, best‐corrected‐visual acuity; logMAR, logarithm of the minimal angle of resolution.

* Chi‐square test was performed with categorical variables and Mann‐Whitney U‐test was performed with continuous variables.

**Figure 2:**
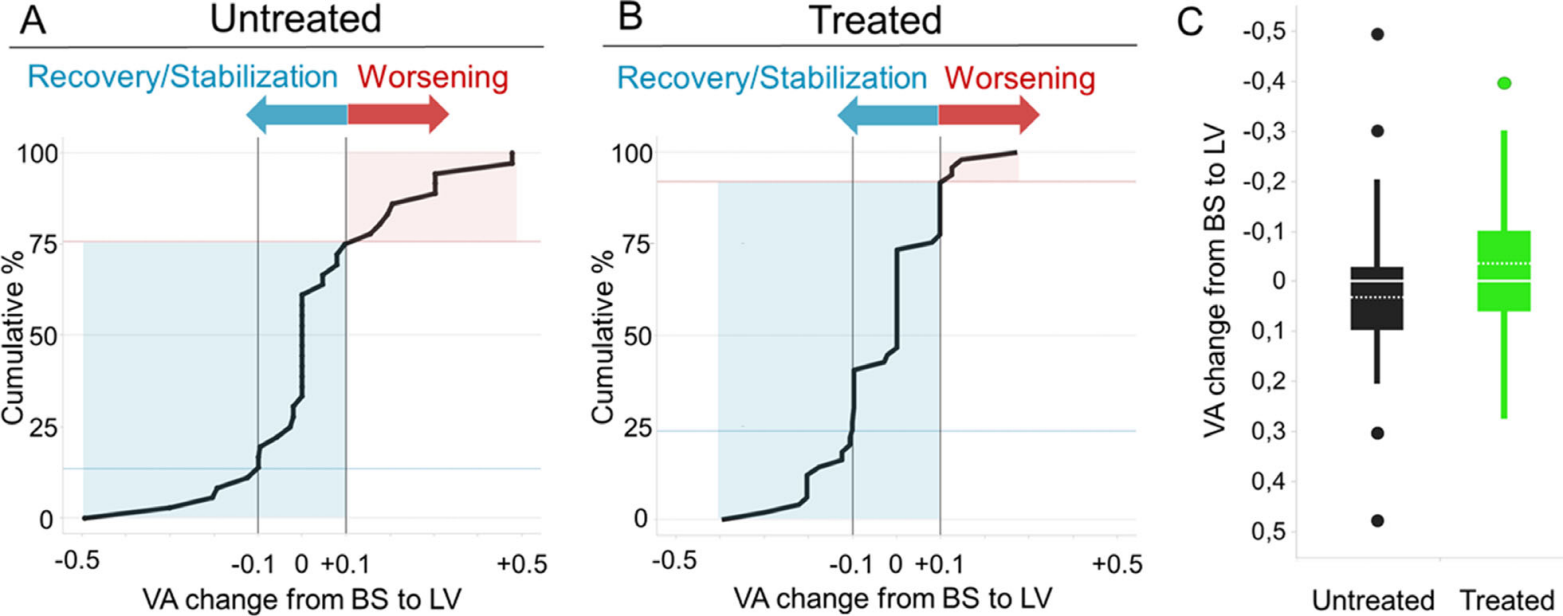
Visual acuity outcome in untreated and idebenone‐treated *OPA1*‐mutant DOA patients. Panels A and B show cumulative frequency graphs of untreated (A) and idebenone‐treated (B) *OPA1*‐DOA patients based on their categorical VA outcome. Light‐blue area represents stable/recovery patients, while pink area corresponds to worsening subgroup of patients. The percentage of idebenone stable/recovery (2B: 92%, *n* = 46) resulted significantly greater than the untreated (2A: 75.7%, *n* = 28) by more than 15% (Chi‐square test, *P* = 0.03). Panel C shows VA change box plot with a solid line representing median value and dotted line representing mean value for both groups. VA = best‐corrected‐visual acuity in logMAR unit; BS = baseline; LV = last visit.

Considering the best‐seeing eyes, the median difference between baseline (VA_untreated_: Mdn, Q1–Q3 = 0.52, 0.2–0.9; VA_idebenone_: Mdn, Q1–Q3 = 0.52, 0.3–1.0) and last visit (VA_untreated_: Mdn, Q1–Q3 = 0.52, 0.3–1.0; VA_idebenone_: Mdn, Q1–Q3 = 0.51, 0.3–0.9) time‐points was significantly different only in the treated group, which showed stability of visual acuity in most cases (*P* = 0.03, see Table S1). Similarly, we found a significant difference between treated and untreated eyes in terms of stable/recovery percentage (Fig. 2A and B), with the same trend for the worst‐seeing eyes (Figure S1). This suggests that idebenone treatment is significantly associated with a favorable outcome. Furthermore, the idebenone treated group showed a smaller change in VA (VA change_untreated_: Mdn, Q1–Q3 = 0.00, −0.04–0.13; VA change_idebenone_: Mdn, Q1–Q3 = 0.00, −0.1–0.08) which further confirms the tendency towards VA‐stabilization in idebenone‐treated group (Mann‐Whitney U‐test, *P* = 0.08) (Fig. 2C).

We also used a univariate logistic regression model (Table 2) to evaluate the relationship between the dichotomous outcome (VA stabilization/recovery/VA worsening) and idebenone treatment, controlling for confounding factors. This analysis showed again that idebenone treatment favored the stabilization/recovery of VA in *OPA1*‐DOA (Table 2).

**Table 2:** Idebenone factor is associated with VA stabilization/recovery in *OPA1*‐mutant DOA patients.

Predictor	Crude Odds Ratio (95% CI)	*P*‐value	Adjusted Odds Ratio (95% CI)	*P*‐value
Idebenone	3.70 (1.04–13.14)	0.043	4.37 (1.01–18.9)	0.049
Gender (male)	0.23 (0.05–1.1)	0.06	0.22 (0.04–1.2)	0.081
Genetics (missense)	1.80 (0.45–7.0)	0.40	1.11 (0.21–5.8)	0.905
VA at baseline (logMAR)	8.60 (1.1–68.3)	0.04	7.40 (0.8–71.1)	0.083
Age at baseline				
12 ≤ Age at baseline < 20^*^, ^1^	1.70 (0.14–21.3)	0.67	0.89 (0.05–15.9)	0.939
Age at baseline> 20^*^, ^1^	0.69 (0.13–3.50)	0.65	0.44 (0.06–3.40)	0.435
Observation time				
2 ≤ Observation time < 6^2^	1.02 (0.20–4.97)	0.98	0.62 (0.10–3.82)	0.611
Observation time> 6^2^	0.30 (0.06–1.40)	0.12	0.16 (0.02–1.02)	0.052

VA, best‐corrected‐visual acuity; logMAR, logarithm of the minimal angle of resolution; CI, Confidence Interval.

^1^ reference: age at baseline < 12 years.

^2^ reference: observation time < 2 years.

## Discussion

The key finding of our study is that off‐label idebenone administration in DOA patients carrying *OPA1* pathogenic mutations was significantly associated with stabilization/recovery of visual acuity. In fact, DOA patients taking idebenone benefit four times more than untreated ones in terms of visual stabilization/recovery, even after controlling for confounders. Despite the major limitations due to the study’s retrospective nature, such as possible sample bias and not‐homogeneous between‐groups observational time and not‐homogeneous idebenone dosage, these findings point to a probable benefit of idebenone therapy for DOA patients.

This study deserves a few comments. Data were collected from both eyes, but we did not combine them using a binary correlation, due to small sample size.^13^, ^14^ Instead, we followed a “one‐eye” approach by analyzing the best‐seeing eyes to run appropriately a logistic regression model and provide valid inference. Our results, based on a relatively large cohort of treated patients and including stability as a positive outcome, highlighted significant differences only for the best‐seeing eyes, with the same trend for the worst‐seeing ones (Figure S1). The reason for this possibly resides in the higher probability that best‐seeing eyes may benefit from idebenone therapy, as they present better preservation of RGCs and axons. Moreover, based on the known natural history of DOA, one advantage, as compared to LHON, is the lack of a clearly documented spontaneous improvement of visual function in DOA.^12^ This allows to more confidently attributing the gain of visual function to idebenone administration. One disadvantage, on the contrary, is the relentless decline of visual function in DOA, frequently characterized by prolonged periods of stability, a factor that lowers the capability to truly and unequivocally detecting therapeutic effectiveness. Overall, these results should be instrumental to prompt a future properly designed double‐blind, placebo‐controlled, randomized trial, to confirm the current observations.

In conclusion, this study strengthens the initial positive trend we have reported in 2013^3^ and reinforces the hypothesis that the complex I defect demonstrated in *OPA1*‐DOA may be ameliorated by idebenone, as for LHON.^2^ Idebenone, in fact, positively modified the natural history of the disease by increasing the chance of stabilization/recovery of vision. The possibility to transfer idebenone therapy from LHON to *OPA1*‐related DOA would represent a very relevant option to fight blindness in the largest categories of inherited optic neuropathies. This is an important opportunity for this rare and currently untreatable disease while waiting for other therapeutic options such as gene therapy^15^ or other strategies under scrutiny^16^ but possibly needing a long way to reach translation into patients.

## Conflict of Interest

The authors declare that they have no conflict of interest related to the content of this article.

## Author Contributions

MR, VC, wrote the first draft of the manuscript. MR, CLM, MC, PB, VC, contributed to the conception and design of the study. MR, CLM, MC, LDV, GA, MLC, PB, VC, contributed to acquisition of the data. MR, CZ, contributed to the statistical analysis of data. MR, CLM, MC, LDV, GA, MLC, PB, VC, contributed to the interpretation of the data. MR, CLM, MC, CZ, PB, VC, contributed to drafting a significant portion of the manuscript or figures or tables. MR, CLM, MC, LDV, GA, CZ, MLC, PB, VC, reviewed the manuscript and provided revisions for intellectual content.

## Supporting information


**Figure S1**. Visual acuity outcome for the worst‐seeing eyes in untreated and idebenone‐treated *OPA1*‐mutant DOA patients.

**Table S1**. Wilcoxon signed‐rank test to compare paired samples, before and after idebenone off‐label administration, in *OPA1*‐mutant DOA patients.
