# Supplementary figures and images for: Idebenone increases chance of stabilization/recovery of visual acuity in OPA1‐dominant optic atrophy
Source: Ann Clin Transl Neurol. 2020 Apr 3;7(4):590–4. doi: 10.1002/acn3.51026 (PMC7187718; doi:10.1002/acn3.51026)

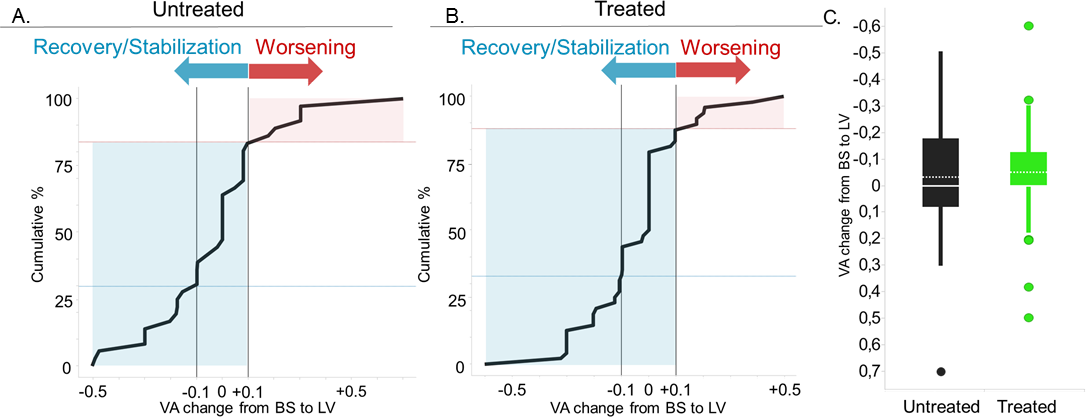

Supplement: Supplementary file 1 — Figure S1. Visual acuity outcome for the worst‐seeing eyes in untreated and idebenone‐treated OPA1‐mutant DOA patients. [file ACN3-7-590-s001.tif]
