# Supplementary material for: Idebenone increases chance of stabilization/recovery of visual acuity in OPA1‐dominant optic atrophy
Source: Ann Clin Transl Neurol. 2020 Apr 3;7(4):590–4. doi: 10.1002/acn3.51026 (PMC7187718; doi:10.1002/acn3.51026)
Supplement: Supplementary file 2 — Table S1. Wilcoxon signed‐rank test to compare paired samples, before and after idebenone off‐label administration, in OPA1‐mutant DOA patients. [file ACN3-7-590-s002.docx]

**Supplementary Table 1. Wilcoxon signed rank test to compare paired samples, before and after idebenone off-label administration, in *OPA1*-mutant DOA patients.**

|  | VA  at baseline | VA  at last visit | p-value |
| --- | --- | --- | --- |
| Best-seeing eyes |  |  |  |
| Untreated  n  Median (Q1-Q3)  95% CI | 37  0.52 (0.22 – 0.9)  0.44-0.72 | 37  0.52 (0.3 – 1)  0.49-0.73 | 0.37 |
| Treated  n  Median (Q1-Q3)  95% CI | 50  0.52 (0.3 -1)  0.55-0.79 | 50  0.51 (0.3 – 0.93)  0.53-0.75 | 0.03 |

Using Wilcoxon signed rank statistics test, it has been possible to analyse whether idebenone off-label administration led overall to a statistically significant difference in VA, paired VA at baseline and at last visit for each subject within treated and untreated groups of *OPA1*-mutant DOA patients. Values are given as n (frequency), median (interquartile range) and 95% CI (Confidence Interval) and are related to VA data expressed in logMAR unit. VA = best-corrected-visual acuity; logMAR = logarithm of the minimal angle of resolution.
